# Glasgow prognostic score for prediction of chemotherapy‐triggered acute exacerbation interstitial lung disease in patients with small cell lung cancer

**DOI:** 10.1111/1759-7714.13900

**Published:** 2021-05-03

**Authors:** Ryota Kikuchi, Hiroyuki Takoi, Takao Tsuji, Yoko Nagatomo, Akane Tanaka, Hayato Kinoshita, Mariko Ono, Mayuko Ishiwari, Kazutoshi Toriyama, Yuta Kono, Yuki Togashi, Kazuhiro Yamaguchi, Akinobu Yoshimura, Shinji Abe

**Affiliations:** ^1^ Department of Respiratory Medicine Tokyo Medical University Hospital Tokyo Japan; ^2^ Respiratory Center Otsuki Municipal Central Hospital Otsuki‐shi Japan; ^3^ Department of Clinical Oncology Tokyo Medical University Hospital Tokyo Japan

**Keywords:** acute exacerbation, Glasgow prognostic score, interstitial lung disease, prognosis, small cell lung cancer

## Abstract

**Background:**

Predicting the incidence of chemotherapy‐triggered acute exacerbation of interstitial lung disease (AE‐ILD) in patients with lung cancer is important because AE‐ILD confers a poor prognosis. The Glasgow prognostic score (GPS), which is an inflammation‐based index composed of serum levels of C‐reactive protein and albumin, predicts prognosis in patients with small cell lung cancer (SCLC) without ILD. In this study, we investigated AE‐ILD and survival outcome based on the GPS in patients with ILD associated with SCLC who were receiving chemotherapy.

**Methods:**

Medical records of patients who received platinum‐based first‐line chemotherapy between June 2010 and May 2019 were retrospectively reviewed to compare the incidence of AE‐ILD and overall survival (OS) between GPS 0, 1, and 2.

**Results:**

Among our cohort of 31 patients, six (19.3%) experienced chemotherapy‐triggered AE‐ILD. The AE‐ILD incidence increased from 9.5% to 25.0% and 50.0% with increase in GPS of 0, 1, and 2, respectively. Univariate and multivariate analyses revealed remarkable associations between GPS 2 and both AE‐ILD (odds ratio for GPS 2, 18.69; *p* = 0.046) and prognosis (hazard ratio of GPS 2, 13.52; *p* = 0.002). Furthermore, median OS in the GPS 0, 1, and 2 groups was 16.2, 9.8, and 7.1 months, respectively (*p* < 0.001).

**Conclusions:**

Our results suggest that GPS 2 is both a predictor of risk of chemotherapy‐triggered AE‐ILD and a prognostic indicator in patients with ILD associated with SCLC. We propose that GPS may be used as a guide to distinguish chemotherapy‐tolerant patients from those at high risk of AE‐ILD.

## Graphical abstract

The Glasgow prognostic score (GPS) tends to be associated with incidence of acute exacerbation of interstitial lung disease (AE‐ILD) (*p* = 0.082). GPS 2 is an independent risk factor for chemotherapy‐triggered AE‐ILD and prognosis in SCLC patients. GPS may be used as a guide to distinguish chemotherapy‐tolerant patients from those at high risk of AE‐ILD.

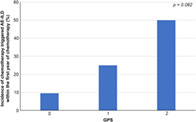

## INTRODUCTION

Patients with interstitial lung disease (ILD) are 7–14 times more prone to lung cancer compared with those without ILD^1^, ^2^, ^3^, ^4^, ^5^; in fact, 4%–38% of patients with ILD concomitantly have lung cancer.^3^, ^4^, ^6^, ^7^, ^8^, ^9^ Therefore, lung cancer is considered the most common complication of ILD. Conversely, ILD is reported in 10%–30% of patients with lung cancer,^10^, ^11^, ^12^, ^13^, ^14^, ^15^ seriously affecting prognosis.^12^, ^16^, ^17^, ^18^, ^19^, ^20^, ^21^ This is because of the limited availability of effective chemotherapy approaches and exacerbation of treatment‐related ILD.^5^, ^18^ Studies exclusively conducted in patients with small cell lung cancer (SCLC) have reported incidence rates of chemotherapy‐triggered acute exacerbation (AE)‐ILD ranging from 11.9% to 36.4%.^22^, ^23^, ^24^, ^25^ These results indicate that lung cancer and ILD occur concomitantly with each other, and it is important to evaluate not only lung cancer but also ILD itself during chemotherapy. However, in most clinical trials, the presence of concomitant ILD is an exclusion criterion for patient enrollment because fatal exacerbation of ILD is problematic. Exacerbation of ILD affects prognosis of lung cancer associated with ILD^5^, ^18^, ^26^, ^27^; therefore, the identification of predictors of ILD exacerbation and prognosis is an important task.

Glasgow prognostic score (GPS) is a score based on systemic inflammation that combines serum albumin (Alb) and serum C‐reactive protein (CRP) levels. GPS was first proposed for non‐small cell lung cancer (NSCLC) by Forrest et al. in 2003 and demonstrated to be a better prognostic factor compared with scoring based on either stage or performance status.^28^ After its introduction, GPS was reported to be a reproducible predictor of long‐term prognosis for NSCLC and SCLC.^29^, ^30^, ^31^, ^32^, ^33^, ^34^, ^35^, ^36^, ^37^, ^38^ However, there have been no previous studies examining the relationship between GPS and SCLC with concomitant ILD in patients undergoing chemotherapy. Therefore, the purpose of this study was to determine whether GPS predicts prognosis and chemotherapy‐triggered AE‐ILD in patients with SCLC and concomitant ILD.

## METHODS

### Patients

The study protocol was approved by the ethical committee of the Tokyo Medical University in Tokyo, Japan (approval number: T2020‐0096). Retrospective data were anonymously analyzed, and patients were given the opportunity to either opt out of the study or provide consent for participation using a waiver of written informed consent granted from the institutional review board. We retrospectively reviewed the medical records of patients with ILD associated with SCLC who received cytotoxic chemotherapy between June 2010 and May 2019 at Tokyo Medical University Hospital. All patients were diagnosed with SCLC cytologically and/or histologically. Tumor, lymph nodes, and distant metastasis (TNM) stage was evaluated based on the eighth edition of TNM classification of lung cancer.^39^ Moreover, patients with SCLC into “limited” disease (LD) or “extensive” disease (ED) were grouped as per the Veterans Administration Lung Study Group staging system.^40^ Physicians selected the chemotherapy regimen considering the therapeutic effect and risk of AE‐ILD; however, patients receiving irinotecan were ineligible because it is a contraindicated chemotherapeutic drug in Japan for patients with SCLC and ILD. Patients who did not receive chemotherapy, transferred to a different hospital before undergoing chemotherapy, or had an infectious condition at first‐line chemotherapy administration, were excluded in this study. Overall survival (OS) was defined as the number of months from first‐line chemotherapy administration until death or censored. We then defined patients as censored if patients were alive on May 31, 2019. Laboratory measurements, including serum CRP and Alb used for GPS, were performed up to one week before first‐line chemotherapy administration. We excluded those patients with SCLC lacking CRP or Alb data. Patients were categorized in the following three GPS‐determined groups: patients with a CRP ≤ 10 mg/l and Alb ≥ 35 g/l were Group 0; patients with a CRP > 10 mg/l or Alb < 35 g/l were Group 1; and patients with a CRP > 10 mg/l and Alb < 35 g/l were Group 2.^28^, ^41^ Moreover, we measured serum lactate dehydrogenase (LDH) and Krebs von den Lungen‐6 (KL‐6) as markers of tissue damage and ILD, respectively.

### Classification of ILD and diagnosis of AE‐ILD

ILD was evaluated based on high‐resolution computed tomography (HRCT) of the chest, which was performed at least 30 days before chemotherapy was first administered. ILD was independently diagnosed by two experienced pulmonologists (R.K. and T.T.) using the criteria of ground‐glass attenuation, reticular shadow, or honeycomb lung in both lung fields, without prior knowledge of patient clinical outcome. Based on recent guidelines for idiopathic pulmonary fibrosis (IPF), we then grouped patients with ILD having an usual interstitial pneumonia (UIP) pattern or non‐UIP pattern.^42^ UIP pattern was then characterized as subpleural and basal dominant reticulation with honeycombing with/without peripheral traction bronchiectasis. The diagnostic criteria for non‐UIP pattern included existence of probable UIP pattern, indeterminate for UIP pattern, or pattern with alternative diagnosis. Chemotherapy‐triggered AE‐ILD was defined as the worsening of dyspnea and newly developed bilateral ground‐glass opacification and/or consolidation within four weeks of the last administration of chemotherapy, not completely explained by infectious disease, cardiac failure, fluid overload, or pulmonary embolism.^43^, ^44^, ^45^, ^46^ We excluded collagen vascular disease‐associated ILD and occupational lung disease because these diseases may cause high levels of CRP and/or low levels of Alb, thereby affecting GPS determination. Our cohort did not include patients who had either received an immunosuppressive drug before chemotherapy or underwent chest radiotherapy and received immunotherapy.

### Statistical analysis

Data from medical records of patients were described either as values (percentages) or as median (range). Among baseline patient characteristics, we analyzed all categorical variables using the Kruskal–Wallis test or chi‐square test as appropriate. Both univariate and multivariate analyses of chemotherapy‐triggered AE‐ILD incidence rate were determined using binomial logistic regression analysis. The Kaplan–Meier method was used to estimate survival outcomes, and log‐rank test was then used to compare the survival of patients using GPS 0, 1, and 2. Both univariate and multivariate analyses with a Cox proportional hazards regression model were used to identify independent risk factors for survival. A *p*‐value < 0.05 was considered statistically significant. All statistical analyses were performed using IBM SPSS Statistics for Windows version 26.0. (IBM Corp.).

## RESULTS

### Patient characteristics

The study flow is summarized in Figure S1. During the study period, eight patients were excluded as follows: two patients diagnosed with secondary ILD (one with rheumatoid arthritis and one with dermatomyositis), serum Alb was not measured in one patient prior to first‐line chemotherapy administration, one patient did not receive chemotherapy, one patient was transferred from our hospital before undergoing first‐line chemotherapy, one patient had an infectious condition, and two patients received irinotecan. Consequently, our cohort comprised 31 patients with ILD associated with SCLC receiving cytotoxic chemotherapy. The baseline characteristics of our patients are shown in Table **1**. The median age of our cohort was 71 years, 26 patients (83.9%) were male, 25 patients (80.6%) had an Eastern Cooperative Oncology Group Performance Status (ECOG PS) of 0 or 1, and 19 patients (61.3%) had TNM lung cancer stage IV. All patients had a smoking history; 15 patients (48.4%) were former smokers and the other 16 (51.6%) were current smokers. A total of 12 patients (38.7%) were classified as LD, whereas 19 patients (61.3%) were ED at the time of first‐line chemotherapy administration. The median serum LDH and KL‐6 levels were 244 U/l and 554 U/ml, respectively. A total of 25 patients (80.6%) underwent a pulmonary function test before administration of chemotherapy; the median predicted forced vital capacity (FVC) was 93.1%. Based on HRCT results, UIP pattern and emphysema were identified in 14 (45.2%) and 26 (83.8%) patients, respectively.

**TABLE 1:** Patient characteristics and clinical manifestation based on Glasgow Prognostic Score (GPS) at time of first‐line chemotherapy

	Total	GPS 0	GPS 1	GPS 2	*p*‐value
Number of patients	31	21	4	6	
Age (years) Median range	71	71 68–78	69 60–77	71 61–75	0.866
Gender (%) Male Female	26 (83.9) 5 (16.1)	18 (85.7) 3 (14.3)	3 (75.0) 1 (25.0)	5 (83.3) 1 (16.7)	0.287
ECOG PS (%) 0, 1 2–4	25 (80.6) 6 (19.4)	16 (76.2) 5 (23.8)	4 (100) 0 (0.00)	5 (83.3) 1 (16.7)	0.534
Smoking history (%) Former Current	15 (48.4) 16 (51.6)	13 (61.9) 8 (38.1)	1 (25.0) 3 (75.0)	1 (16.7) 5 (83.3)	0.089
Pack‐years Median range	50.0	60.0 40.0–85.0	45.0 24.2–50.0	41.5 23.2–54.2	0.054
BMI (kg/m^2^) Median range	22.5	22.4 21.2–24.3	24.3 20.5–26.2	22.7 18.1–24.2	0.610
Disease stage (%) Limited Extensive	12 (38.7) 19 (61.3)	10 (47.6) 11 (52.4)	1 (25.0) 3 (75.0)	1 (16.7) 5 (83.3)	0.325
Clinical stage (%) III IV	12 (38.7) 19 (61.3)	6 (19.4) 25 (80.6)	1 (25.0) 3 (75.0)	2 (33.3) 4 (66.7)	0.763
LDH (U/l) Median range	244	236 209–293	299 218–829	254 200–670	0.521
KL‐6 (U/ml) Median range	554	554 369–933	860 288–1330	547 303–709	0.701
% predicted FVC Median range	93.1	101.9 (*n* = 17) 90.3–116.2	92.5 (*n* = 4) 77.9–100.1	80.4 (*n* = 4) 52.9–89.9	0.027
HRCT pattern (%) UIP pattern Non‐UIP pattern	14 (45.2) 17 (54.8)	10 (47.6) 11 (52.4)	2 (50.0) 2 (50.0)	2 (33.3) 4 (66.7)	0.807
Emphysema (%) Yes No	26 (83.8) 5 (16.1)	18 (85.7) 3 (14.3)	4 (100.0) 0 (0.00)	5 (83.3) 1 (16.6)	0.703
Second‐line chemotherapy (%)	20 (64.5)	13 (61.9)	3 (75.0)	4 (66.6)	0.267
Third‐line chemotherapy (%)	5 (16.1)	3 (14.2)	0 (0.00)	2 (33.3)	—

Abbreviations: BMI, body mass index; ECOG PS, Eastern Cooperative Oncology Group Performance Status; FVC, forced vital capacity; HRCT, high‐resolution computed tomography; KL‐6, Krebs von den Lungen‐6; LDH, lactate dehydrogenase; UIP, usual interstitial pneumonia.

### Association of GPS with clinicopathological parameters

Based on GPS, 21 (67.7%), four (12.9%), and six (19.3%) patients were classified to the GPS 0, 1, and 2 groups, respectively. Patient characteristics in GPS subgroups are shown in Table 1. We reported no significant differences among the three groups except for decreased predicted FVC with higher GPS (*p* = 0.027).

### Chemotherapy‐triggered AE‐ILD

**FIGURE 1:**
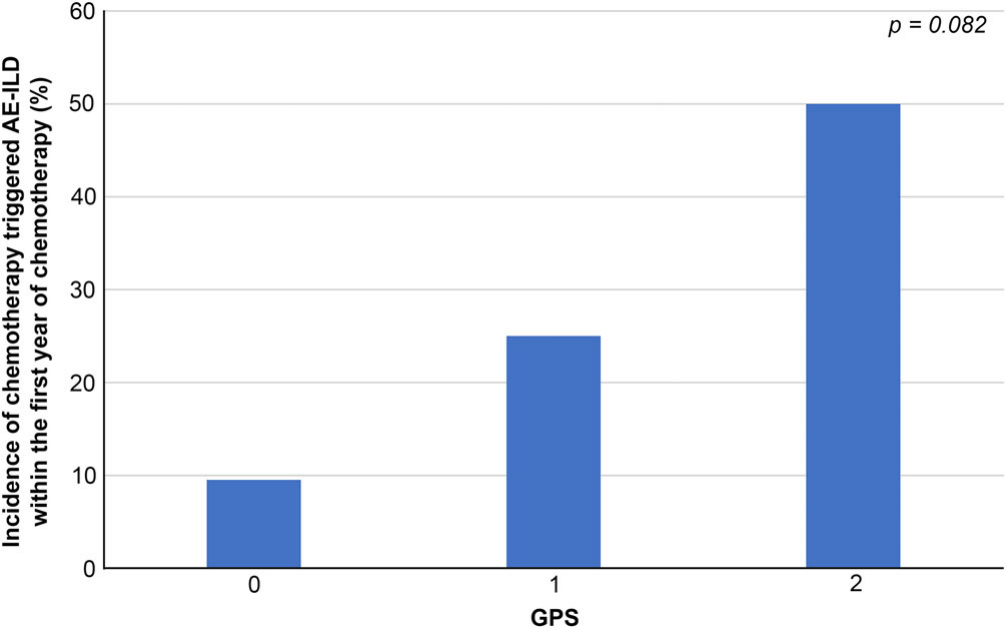
Incidence of chemotherapy‐triggered acute exacerbation of interstitial lung disease (AE‐ILD) in patients with small cell lung cancer (SCLC) according to the Glasgow prognostic score (GPS). The patient subgroups were as follows: GPS 0, *n* = 21; GPS 1, *n* = 4; and GPS 2, *n* = 6

During the present study period, cumulative incidence of chemotherapy‐triggered AE‐ILD is shown in Table S1. Of our 31 patients, six experienced chemotherapy‐triggered AE‐ILD and none experienced more than two episodes of AE‐ILD. The evaluation of patients who experienced AE showed that two patients had a GPS of 0, one patient had a score of 1, and three patients had a GPS of 2. All patients developed grade ≥3 pneumonitis, and one patient died of chemotherapy‐triggered AE‐ILD. All patients experienced AE‐ILD within a year of receiving cytotoxic chemotherapy. Based on our evaluation, the annual incidence of chemotherapy‐triggered AE‐ILD in patients with GPS 0, 1, and 2 was 9.5%, 25.0%, and 50.0%, respectively (Figure 1). Furthermore, our results indicate that GPS tended to be associated with incidence of AE‐ILD (*p* = 0.082). Next, we determined whether there was evidence of a relationship between incidence of AE‐ILD and individual variables (shown in Table 2). Based on univariate logistic regression analysis, LDH, HRCT pattern, and GPS were selected as candidate risk factors having *p* < 0.2. We excluded predicted FVC from analysis because of incomplete cohort data. Using multivariate logistic regression analysis, we reported that GPS 2 was the only predictor significantly associated with incidence of chemotherapy‐triggered AE‐ILD (GPS 2 odds ratio [OR], 18.69; 95% confidence interval [CI]: 1.04–333.49; *p* = 0.046). Although LDH and HRCT pattern exhibited a trend toward association with incidence of AE‐ILD, the results were not statistically significant (LDH OR, 0.98; 95% CI: 0.95–1.00; *p* = 0.19 | HRCT OR, 0.81; 95% CI: 0.049–13.68; *p* = 0.88). Chemotherapy regimens are shown in Table S2. In our study, all patients received cisplatin or carboplatin and etoposide for first‐line treatment. The incidence of AE‐ILD by first‐ and second−/third‐line chemotherapy was 6.4% (two patients) and 16% (four patients), respectively. The frequency of chemotherapy‐triggered AE‐ILD according to each cytotoxic chemotherapy regimen is summarized in Table S3. We reported that the incidence of AE‐ILD in topotecan‐ and amrubicin‐treated groups was 25% (two patients) and 25% (one patient) respectively, both of which are higher than that reported of other drugs (Table S3).

**TABLE 2:** Univariate and multivariate analyses of factors associated with chemotherapy‐triggered acute exacerbation of interstitial lung disease (AE‐ILD) in patients with small cell lung cancer (SCLC) and interstitial lung disease (ILD) (*n* = 31)

Variable	Univariate analysis			Multivariate analysis		
	OR	95% CI	*p*‐value	OR	95% CI	*p*‐value
Age, per year	0.97	0.86–1.10	0.71			
Gender (male vs. female)	3.66	0.45–29.41	0.22			
ECOG PS (0, 1 vs. 2–4)	2.62	0.35–19.51	0.34			
Smoking history (former vs. current)	0.39	0.06–2.55	0.32			
Pack‐years, per pack‐year	0.98	0.94–1.02	0.42			
BMI, per kg/m^2^	0.86	0.58–1.27	0.47			
Disease stage (Limited vs. Extensive)	1.33	0.20–8.70	0.76			
Clinical stage (III vs. IV)	1.33	0.20–8.70	0.76			
LDH, per U/l	0.98	0.96–1.00	0.14	0.98	0.95–1.00	0.19
KL‐6, per U/ml	0.99	0.99–1.00	0.31			
HRCT pattern (non‐UIP vs. UIP pattern)	0.18	0.019–1.81	0.14	0.81	0.049–13.68	0.88
Emphysema (No/Yes)	0.68	0.058–8.00	0.76			
GPS						
0	1 (Ref)			1 (Ref)		
1	3.16	0.21–46.72	0.40	6.87	0.27–175.27	0.24
2	9.50	1.09–82.72	0.041	18.69	1.04–333.49	0.046

Abbreviations: AE, acute exacerbation; BMI, body mass index; CI, confidence interval; ECOG PS, Eastern Cooperative Oncology Group Performance Status; GPS, Glasgow prognostic score; HRCT, high‐resolution computed tomography; KL‐6, Krebs von den Lungen‐6; LDH, lactate dehydrogenase; OR, odds ratio; Ref, reference; UIP, usual interstitial pneumonia.

### Prognosis

**FIGURE 2:**
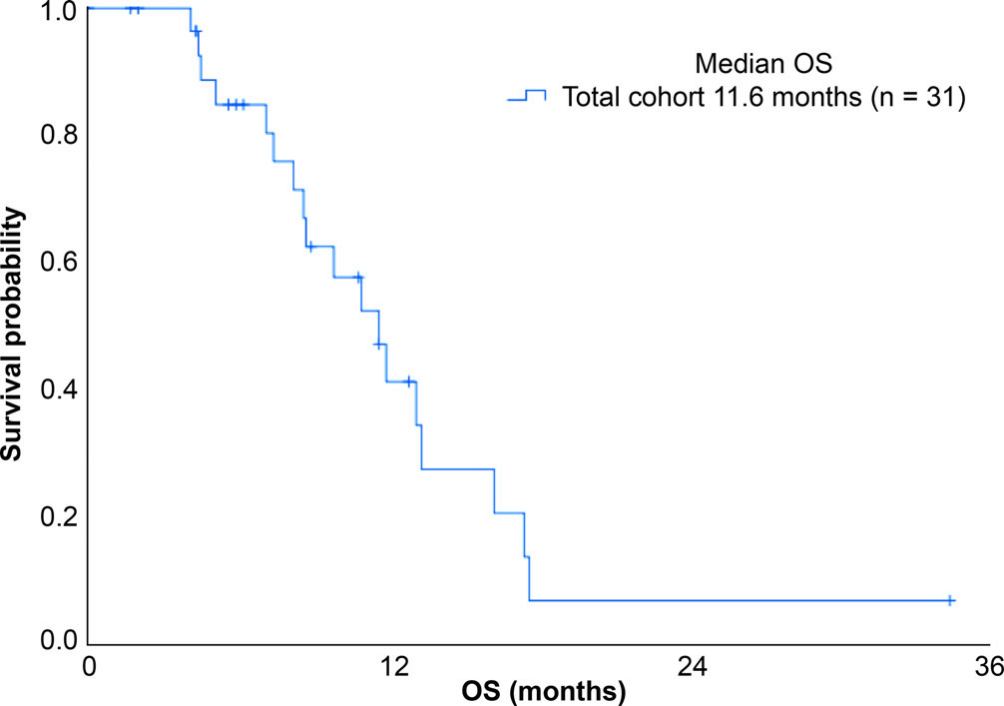
Overall survival (OS) rate of patients with interstitial lung disease (ILD) associated with small cell lung cancer (SCLC) receiving chemotherapy (*n* = 31)

**FIGURE 3:**
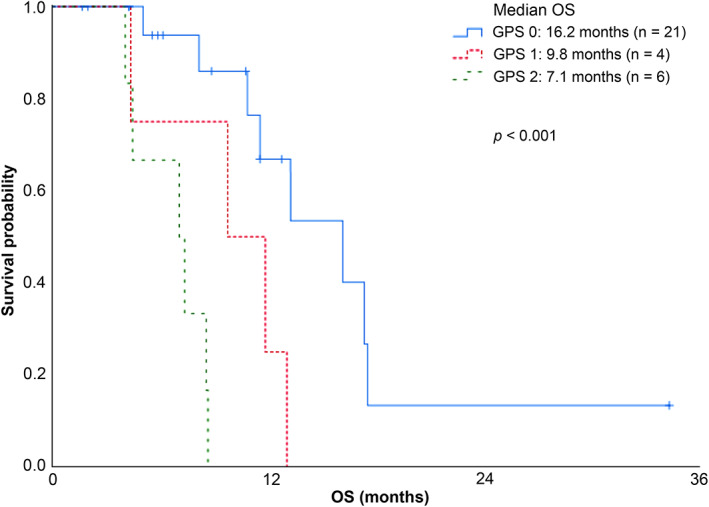
Overall survival (OS) rate of patients with interstitial lung disease (ILD) associated with SCLC receiving chemotherapy based on the Glasgow prognostic score (GPS) group. Patient subgroups were as follows: GPS 0, *n* = 21; GPS 1, *n* = 4; and GPS 2, *n* = 6

Among all patients in our cohort, we reported one‐year survival and median OS of 44% and 11.6 months, respectively (Figure 2). We then compared median OS according to GPS and reported median OS of 16.2, 9.8, and 7.1 months in patients with GPS 0, 1, and 2, respectively (*p* < 0.001; Figure 3). These results indicate that GPS is significantly related with prognosis in patients with ILD associated with SCLC receiving chemotherapy. Next, we determined whether there was a relationship between median OS and individual variables (Table 3). Based on univariate Cox proportional hazards regression analysis, we reported that serum LDH level and GPS 2 were significantly correlated with median OS (LDH hazard ratio [HR], 1.00; 95% CI: 1.00–1.00; *p* = 0.004 | GPS 1 HR, 3.98; 95% CI: 0.99–16.00; *p* = 0.051 | GPS 2 HR, 17.29; 95% CI: 3.58–83.35; *p* < 0.001). Following multivariate analyses, we reported that only GPS 2 was independently associated with prognosis after adjusting for LDH and GPS 1 (LDH HR, 1.00; 95% CI: 0.99–1.00; *p* = 0.11 | GPS 1 HR, 3.76; 95% CI: 0.93–15.15; *p* = 0.062 | GPS 2 HR, 13.52; 95% CI: 2.59–70.41; *p* = 0.002). Moreover, we performed the subgroup analysis of survival curve in patients with ED‐SCLC. Among these 19 patients, we determined one‐year survival and median OS of 38% and 9.8 months, respectively (Figure S2). Further, median OS of patients with GPS 0, 1, and 2 was 13.3, 9.8, and 7.4 months, respectively (*p* < 0.004; Figure S3).

**TABLE 3:** Univariate and multivariate analyses of factors associated with overall survival (OS) in patients with small cell lung cancer (SCLC) and interstitial lung disease (ILD) (*n* = 31)

Variable	Univariate analysis			Multivariate analysis		
	HR	95% CI	*p*‐value	HR	95% CI	*p*‐value
Age, per year	1.01	0.93–1.09	0.80			
Gender (male vs. female)	0.73	0.16–3.28	0.68			
ECOG PS (0, 1 vs. 2–4)	2.65	0.56–12.45	0.21			
Smoking history (former vs. current)	1.85	0.71–4.84	0.20			
Pack‐years, per pack‐year	1.00	0.98–1.01	0.89			
BMI, per kg/m^2^	1.00	0.80–1.25	0.95			
Disease stage (Limited vs. Extensive)	2.28	0.77–6.72	0.13			
Clinical stage (III vs. IV)	1.56	0.55–4.39	0.39			
LDH, per U/l	1.00	1.00–1.00	0.004	1.00	1.00–1.00	0.11
KL‐6, per U/ml	1.00	0.99–1.00	0.99			
HRCT pattern (non‐UIP vs. UIP pattern)	0.97	0.38–2.49	0.95			
Emphysema (No/Yes)	1.68	0.44–6.37	0.44			
GPS						
0	1 (Ref)			1 (Ref)		
1	3.98	0.99–16.00	0.051	3.76	0.93–15.15	0.062
2	17.29	3.58–83.35	<0.001	13.52	2.59–70.41	0.002

Abbreviations: BMI, body mass index; CI, confidence interval; ECOG PS, Eastern Cooperative Oncology Group Performance Status; GPS, Glasgow prognostic score; HR, hazard ratio; HRCT, high‐resolution computed tomography; KL‐6, Krebs von den Lungen‐6; LDH, lactate dehydrogenase; Ref, reference; UIP, usual interstitial pneumonia.

## DISCUSSION

We believe that this is the first study to investigate whether there is a relationship between GPS and patients with SCLC and ILD receiving chemotherapy. The results show that GPS 2 is a significant predictive factor for the incidence of chemotherapy‐triggered AE‐ILD; furthermore, we reported that GPS was associated with OS in patients with SCLC and ILD.

The annual incidence of AE‐ILD in patients with GPS 2 was 50.0% (three patients), which was higher than that reported ILD associated with SCLC who received cytotoxic chemotherapy (11.9%–36.4%).^22^, ^23^, ^24^, ^25^ However, the frequency of AE‐ILD within the first year of chemotherapy of 9.5% (two patients) in the GPS 0 subgroup was lower than that previously reported. Based on these results, we propose that using GPS to monitor patients could lead to mitigation of chemotherapy‐triggered AE‐ILD risk. Importantly, in a clinical setting, GPS may provide guidance to identify patients tolerant of chemotherapy from those at high risk of AE‐ILD.

Previously, studies reported a median OS ranging from 9.4 to 10.6 months using platinum‐based anticancer agents and etoposide as first‐line chemotherapy for patients with ED‐SCLC without ILD.^47^, ^48^, ^49^ In our study, the median OS of patients with ED‐SCLC and GPS 0 was 13.3 months, which is comparable to previous results. These data suggest that the risk for ILD exacerbation is low and that chemotherapy is warranted for patients with SCLC and a GPS of 0. Meanwhile, we reported that the median OS of patients with ED‐SCLC and GPS 2 was 7.4 months, a result that indicates worse prognosis than that reported in previous studies. As GPS 2 is a significant predictor of chemotherapy‐triggered AE‐ILD, the poorer prognosis of patients with a GPS of 2 is possibly attributable to AE‐ILD. Furthermore, the incidence of AE in second‐line and later treatments was higher than that reported in first‐line treatment (Figure S1). Because there are no established methods currently available for treating chemotherapy‐triggered AE‐ILD and with a mortality of ILD exacerbation between 22% and 27%,^50^, ^51^, ^52^, ^53^, ^54^ patients with ED‐SCLC and ILD with a GPS of 2 should receive adequate explanation of risks before receiving chemotherapy and availability of other options to consider such as supportive care especially in second‐line or later treatments. Furthermore, caution should be exercised with the use of topotecan because it was associated with an AE incidence of 25%, which is similar to that reported in a previous study.^55^ Conversely, carboplatin and paclitaxel are associated with lower AE incidence rates, thus warranting consideration of their use.

The mechanism underlying the prognostic effect of GPS in patients with ILD associated with lung cancer remains unclear. One possible explanation for the association between high levels of GPS and prognosis is that systemic inflammation and/or malnutrition are contributory factors in chemotherapy‐triggered AE‐ILD. It has been reported that a high serum CRP level, reflecting a state of inflammation, is closely related to AE‐ILD.^56^, ^57^, ^58^, ^59^, ^60^, ^61^, ^62^, ^63^, ^64^ Zhuang et al. suggested that elevated serum CRP was a prognostic factor of hospital mortality in patients with AE‐IPF.^63^ Furthermore, Minegishi et al. proposed the high levels of CRP as a predictive factor for AE‐ILD in patients with ILD associated with lung cancer treated using chemotherapy.^56^ Meanwhile, Alb is a negative‐phase protein used as a marker of inflammation and nutrition, which has been reported to be associated with mortality of patients with AE‐IPF and patients with ILD awaiting transplant.^58^, ^65^ Moreover, Biyun et al. demonstrated that IPF patients with hypoprealbuminemia have poorer outcome.^66^ In our study, GPS 2 is associated with chemotherapy‐triggered AE‐ILD and prognosis in patients with SCLC and pre‐existing ILD, thus supporting previous studies. Furthermore, it was proposed that the treatment of inflammation and undernutrition may be effective for preventing AE‐ILD and confer improved prognosis for patients with SCLC and ILD.

Some clinical factors, such as age, ECOG PS, and disease stage, are known to impact survival in SCLC patients without ILD.^67^, ^68^, ^69^ However, in the present study, these factors did not prove to be significant prognostic factors. This could be explained by the involvement of ILD. Some studies have suggested that exacerbation of ILD has been associated with the prognosis of SCLC patients with ILD.^26^, ^27^ In our study, after adjustment for LDH, multivariate analyses revealed that AE‐ILD was a prognostic factor (Table S4). However, age, ECOG PS, and disease stage were not significant predictive factors for incidence of AE‐ILD, which suggests that these factors may fail to predict prognosis. Furthermore, radiotherapy has not been routinely used for the treatment of SCLC patients with ILD, regardless of whether patients are young, have a favorable PS, or have a SCLC classified as a LD. Standard therapy could not be performed in these patients, which may explain the absence of an association between their prognosis and age, EOCG PS, and disease stage. Consistent with the results of our study, previous studies did not find these factors to be significant prognosis predictors in SCLC patients with ILD.^26^, ^70^ As such, further studies will be required to evaluate the usefulness of clinical factors and GPS as prognostic predictors in SCLC patients with/without ILD.

Our study has several limitations. First, this was a nonrandomized retrospective study conducted in a small number of patients, which may have resulted in various biases. However, to date, no large‐scale phase 3 studies of pharmacotherapy have been conducted in SCLC patients with ILD. Given the difficulty of conducting prospective studies in this area, our study remains important despite its retrospective nature and small sample size. Furthermore, previous studies had sample sizes ranging from 17 to 59 patients,^22^, ^24^, ^25^, ^26^, ^27^, ^43^ and many were as large as our study. Even with such a small sample size, our study identified GPS as a significant marker for predicting the occurrence of AE‐ILD and the prognosis of patients with SCLC and ILD. In the future, further large‐scale prospective studies are warranted to validate our findings. Second, we had only a single patient with LD‐SCLC and a GPS of 1 or 2, respectively. Therefore, we were unable to analyze prognostic differences depending on GPS in patients with LD‐SCLC. However, our study revealed an inverse relationship between GPS and median OS in patients with ED‐SCLC and ILD. Third, we were unable to include predicted FVC as a variable in logistic regression analyses with the incidence of AE‐ILD because six patients in our cohort did not undergo a pulmonary function test; this may introduce selection bias in our results. However, there was no evidence that predicted FVC was a significant predictor of AE‐ILD in those patients only who underwent pulmonary function testing before first‐line chemotherapy (Table S5).

Our study provides evidence of previously unreported associations between GPS and chemotherapy‐triggered AE‐ILD and prognosis in patients with ILD associated with SCLC. We demonstrated that high GPS serves as a candidate predictor for AE‐ILD development. Thus, GPS may provide clinical guidance for identifying patients tolerant of chemotherapy from those with higher risk of treatment‐related complications particularly AE‐ILD; consequently, GPS may predict the prognosis for patients with ILD associated with SCLC.

## CONFLICT OF INTEREST

The authors declare no competing interests.

## Supporting information


**Figure S1**. Flowchart of patient enrollment. SCLC, small cell lung cancer; ILD, interstitial lung disease; GPS, Glasgow prognostic score

**Figure S2**. Overall survival rate of patients with ILD associated with ED‐SCLC who received chemotherapy (*n* = 19). OS, overall survival; ILD, interstitial lung disease; ED, extensive disease; SCLC, small cell lung cancer

**Figure S3**. Overall survival rate of patients with ILD associated with ED‐SCLC who received chemotherapy according to GPS. Patient subgroups were as follows: GPS 0, *n* = 11; GPS 1, n = 3; and GPS 2, *n* = 5). OS, overall survival; ILD, interstitial lung disease; ED, extensive disease; SCLC, small cell lung cancer; GPS, Glasgow prognostic score

**Table S1**. Characteristics of patients who developed chemotherapy triggered AE‐ILD (*n* = 6)
**Table S2**. Frequency of chemotherapy regimens and incidence rate of chemotherapy triggered AE‐ILD related to first‐, second‐, and third‐line regimens
**Table S3**. Incidence of chemotherapy triggered AE‐ILD for different chemotherapy regimens
**Table S4**. Univariate and multivariate analyses of LDH and AE‐ILD associated with overall survival in patients with SCLC and ILD (*n* = 31)
**Table S5**. Univariate analysis of factors associated with chemotherapy triggered AE‐ILD in patients with SCLC and ILD who underwent pulmonary function test (*n* = 25)
