# Supplementary figures and images for: Glasgow prognostic score for prediction of chemotherapy‐triggered acute exacerbation interstitial lung disease in patients with small cell lung cancer
Source: Thorac Cancer. 2021 May 3;12(11):1681–9. doi: 10.1111/1759-7714.13900 (PMC8169307; doi:10.1111/1759-7714.13900)

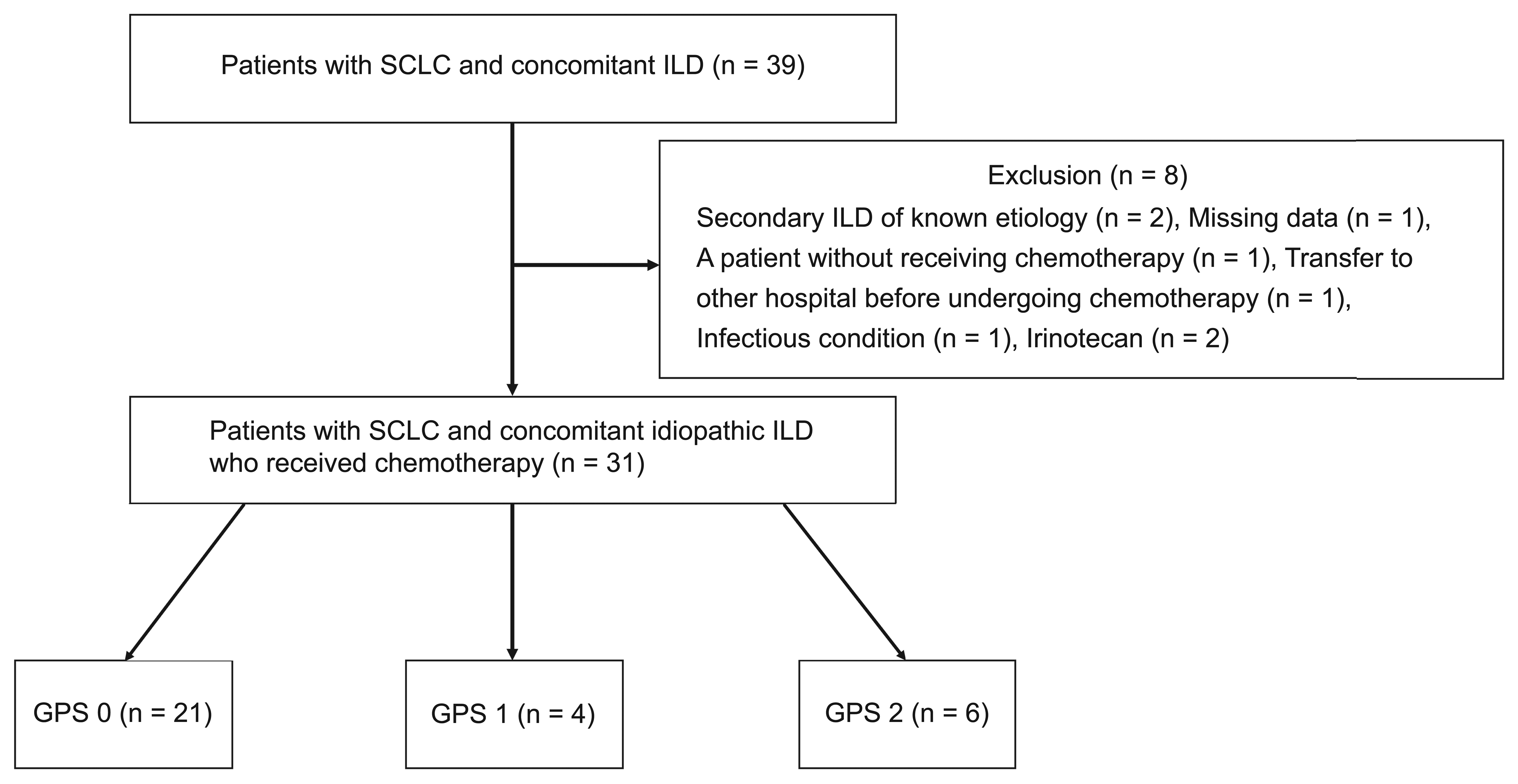

Supplement: Supplementary file 1 — Figure S1. Flowchart of patient enrollment. SCLC, small cell lung cancer; ILD, interstitial lung disease; GPS, Glasgow prognostic score [file TCA-12-1681-s002.tif]

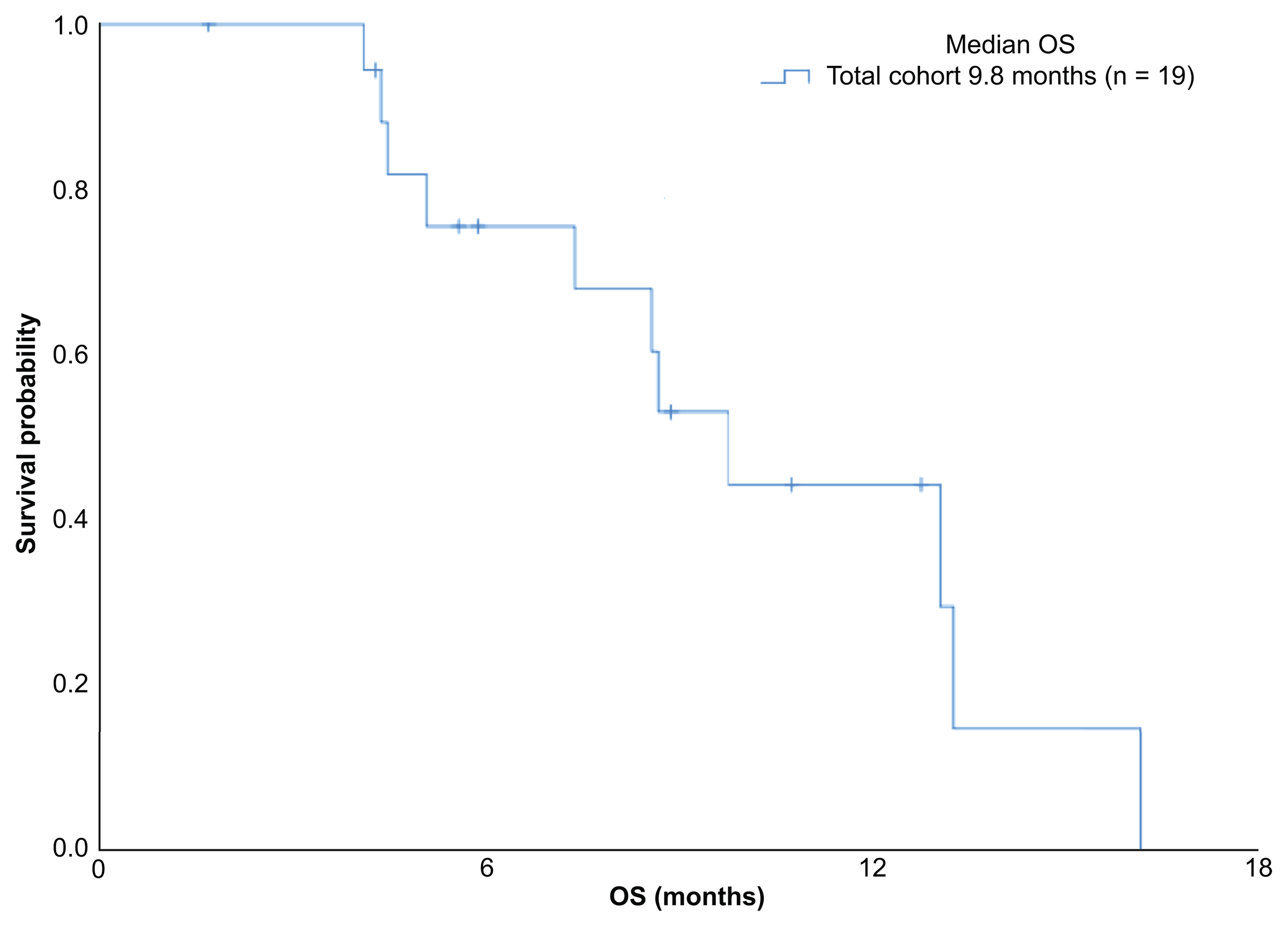

Supplement: Supplementary file 2 — Figure S2. Overall survival rate of patients with ILD associated with ED‐SCLC who received chemotherapy (n = 19). OS, overall survival; ILD, interstitial lung disease; ED, extensive disease; SCLC, small cell lung cancer [file TCA-12-1681-s003.tif]

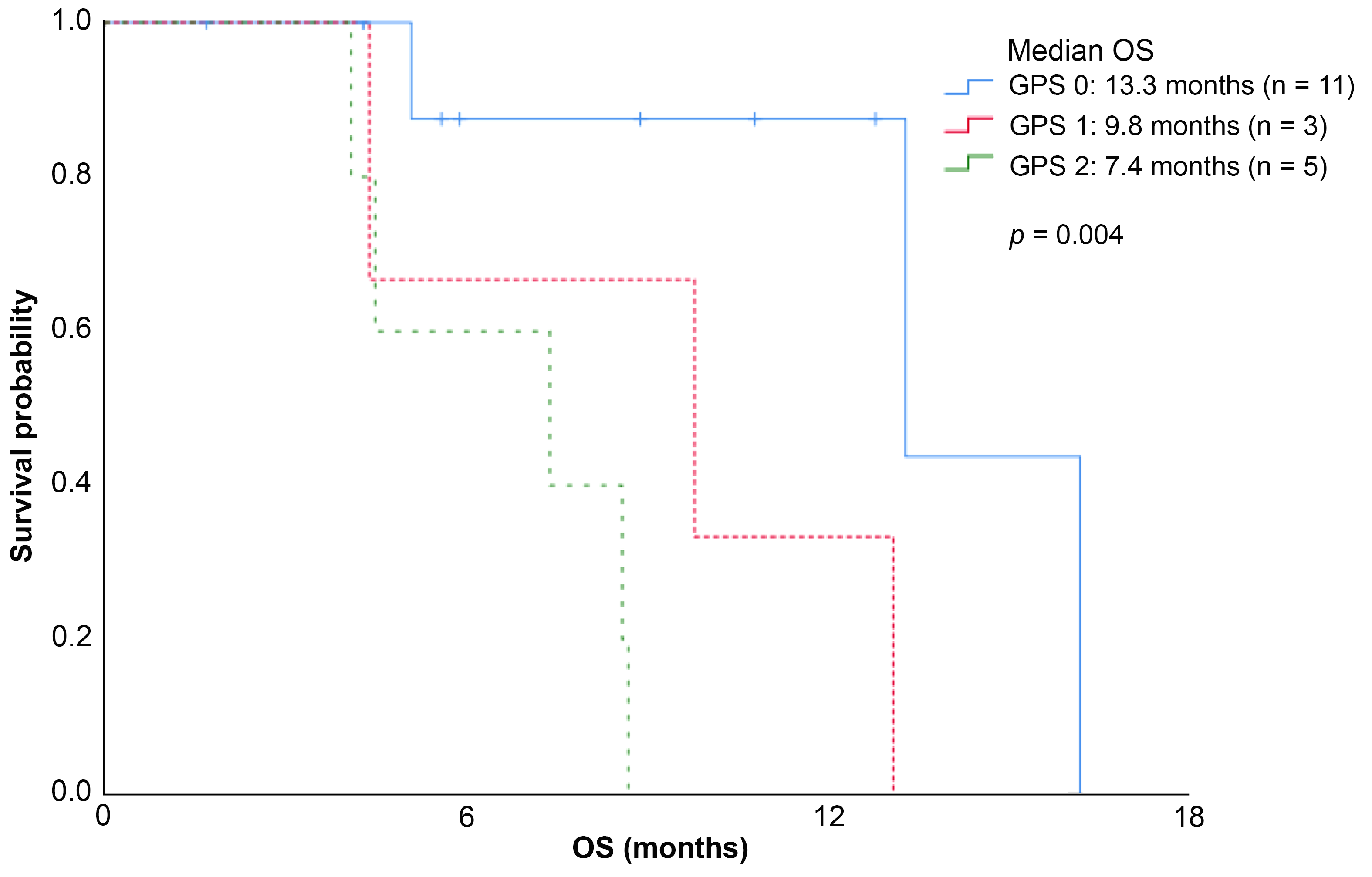

Supplement: Supplementary file 3 — Figure S3. Overall survival rate of patients with ILD associated with ED‐SCLC who received chemotherapy according to GPS. Patient subgroups were as follows: GPS 0, n = 11; GPS 1, n = 3; and GPS 2, n = 5). OS, overall survival; ILD, interstitial lung disease; ED, extensive disease; SCLC, small cell lung cancer; GPS, Glasgow prognostic score [file TCA-12-1681-s004.tif]
