# Supplementary material for: Glasgow prognostic score for prediction of chemotherapy‐triggered acute exacerbation interstitial lung disease in patients with small cell lung cancer
Source: Thorac Cancer. 2021 May 3;12(11):1681–9. doi: 10.1111/1759-7714.13900 (PMC8169307; doi:10.1111/1759-7714.13900)
Supplement: Supplementary file 4 — Table S1. Characteristics of patients who developed chemotherapy triggered AE‐ILD (n = 6) Table S2. Frequency of chemotherapy regimens and incidence rate of chemotherapy triggered AE‐ILD related to first‐, second‐, and third‐line regimens Table S3. Incidence of chemotherapy triggered AE‐ILD for different chemotherapy regimens Table S4. Univariate and multivariate analyses of LDH and AE‐ILD associated with overall survival in patients with SCLC and ILD (n = 31) Table S5. Univariate analysis of factors associated with chemotherapy triggered AE‐ILD in patients with SCLC and ILD who underwent pulmonary function test (n = 25) [file TCA-12-1681-s001.docx]

# Supporting information

**Fig S1.** **Flowchart of patient enrollment**

SCLC, small-cell lung cancer; ILD, interstitial lung disease; GPS, Glasgow prognostic score

**Fig S2. Overall survival rate of patients with ILD associated with ED-SCLC who received chemotherapy (n = 19)**

OS, overall survival; ILD, interstitial lung disease; ED, extensive disease; SCLC, small cell lung cancer

**Fig S3. Overall survival rate of patients with ILD associated with ED-SCLC who received chemotherapy according to GPS**

Patient subgroups were as follows: GPS 0, n = 11; GPS 1, n = 3; and GPS 2, n = 5). OS, overall survival; ILD, interstitial lung disease; ED, extensive disease; SCLC, small-cell lung cancer; GPS, Glasgow prognostic score

**Table S1. Characteristics of patients who developed chemotherapy triggered AE-ILD (n = 6)**

**Table S2. Frequency of chemotherapy regimens and incidence rate of chemotherapy triggered AE-ILD related to first-, second-, and third-line regimens**

**Table S3. Incidence of chemotherapy triggered AE-ILD for different chemotherapy regimens**

**Table S4. Univariate and multivariate analyses of LDH and AE-ILD associated with overall survival in patients with SCLC and ILD (n = 31)**

**Table S5. Univariate analysis of factors associated with chemotherapy triggered AE-ILD in patients with SCLC and ILD who underwent pulmonary function test (n = 25)**

**Table S1. Characteristics of patients who developed chemotherapy triggered AE-ILD (n = 6)**

| **Patient** | **Age (years)** | **Gender** | **PS** | **Disease**  **stage** | **% predicted FVC** | **HRCT pattern** | **GPS** | **Line** | **Chemotherapy before AE-ILD** | **Grade** | **OS**  **(months)** |
| --- | --- | --- | --- | --- | --- | --- | --- | --- | --- | --- | --- |
| **1** | 80 | Male | 2 | ED | 86.4 | Non-  UIP | 2 | 1 | CBDCA+ETP | 5 | 4.1 |
| **2** | 70 | Male | 1 | LD | 74.5 | Non-  UIP | 2 | 1 | CBDCA+ETP | 3 | 7.1 |
| **3** | 68 | Male | 0 | ED | 120.1 | Non-  UIP | 0 | 2 | TOPO | 3 | 5.6 |
| **4** | 71 | Male | 2 | LD | 93.1 | UIP | 0 | 2 | TOPO | 3 | 10.9 |
| **5** | 55 | Female | 0 | ED | 45.8 | Non-  UIP | 2 | 2 | AMR | 3 | 7.4 |
| **6** | 79 | Female | 1 | ED | 96.4 | Non-  UIP | 1 | 2 | CBDCA+PTX | 3 | 13.1 |

AE, acute exacerbation; ILD, interstitial lung disease; PS, performance status; FVC, forced vital capacity; HRCT, high-resolution computed tomography; GPS, Glasgow prognostic score; OS, overall survival; ED, extensive disease; LD, limited disease; UIP, usual interstitial pneumonia; CBDCA, carboplatin; ETP, etoposide; TOPO, topotecan; AMR, amrubicin; PTX, paclitaxel.”

**Table S2. Frequency of chemotherapy regimens and incidence rate of chemotherapy triggered AE-ILD related to first-, second-, and third-line regimens**

|  | **Regimen** | **# of Patients** | **AE** |
| --- | --- | --- | --- |
| **First line** | CDDP/CBDCA+ETP | 31 (100%) | 2 (6.4%) |
| **Total** |  | 31 | 2 (6.4%) |
| **Second and Third line** | CBDCA+PTX | 11 (45%) | 1 (9.0%) |
|  | TOPO | 8 (35%) | 2 (25.0%) |
|  | AMR | 4 (10%) | 1 (25.0%) |
|  | CDDP/CBDCA+ETP | 2 (10%) | 0 (0%) |
| **Total** |  | 25 | 4 (16%) |

AE, acute exacerbation; ILD, interstitial lung disease; CDDP, cisplatin; CBDCA, carboplatin; ETP, etoposide; PTX, paclitaxel; TOPO, topotecan; AMR, amrubicin

**Table S3. Incidence of chemotherapy triggered AE-ILD for different chemotherapy regimens**

| **Chemotherapy** | **Incidence of AE** |
| --- | --- |
| Platinum agents (n = 44) | 2 (4.5%) |
| ETP (n = 33) | 2 (6.0%) |
| PTX (n = 11) | 1 (9.0%) |
| TOPO (n = 8) | 2 (25%) |
| AMR (n = 4) | 1 (25%) |

AE, acute exacerbation; ILD, interstitial lung disease; n, number; ETP, etoposide; PTX, paclitaxel; TOPO, topotecan; AMR, amrubicin

**Table S4. Univariate and multivariate analyses of LDH and AE-ILD associated with overall survival in patients with SCLC and ILD (n = 31)**

| **Variable** | **Univariate analysis** |  |  | **Multivariate analysis** |  |  |
| --- | --- | --- | --- | --- | --- | --- |
|  | **HR** | **95% CI** | ***p*** | **HR** | **95% CI** | ***p*** |
| **LDH, per U/L** | 1.00 | 1.00-  1.00 | 0.004 | 1.00 | 1.00-  1.00 | 0.001 |
| **AE-ILD (No vs. Yes)** | 2.84 | 0.94-  8.56 | 0.064 | 3.96 | 1.23-  12.70 | 0.021 |

LDH, lactate dehydrogenase; AE, acute exacerbation; ILD, interstitial lung disease; SCLC, small-cell lung cancer; HR, hazard ratio; CI, confidence interval.

**Table S5. Univariate analysis of factors associated with chemotherapy triggered AE-ILD in patients with SCLC and ILD who underwent pulmonary function test (n = 25)**

| **Variable** | **OR** | **95% CI** | ***p*** |
| --- | --- | --- | --- |
| **% predicted FVC** | 0.959 | 0.905–1.105 | 0.150 |

AE, acute exacerbation; ILD, interstitial lung disease; SCLC, small-cell lung cancer; OR, odds ratio; CI, Confidence interval; FVC, forced vital capacity
